# Performance evaluation of image co-registration methods in photoacoustic mesoscopy of the vasculature

**DOI:** 10.1088/1361-6560/ad7fc7

**Published:** 2024-10-17

**Authors:** T L Lefebvre, P W Sweeney, J Gröhl, L Hacker, E L Brown, T R Else, M-E Oraiopoulou, A Bloom, D Y Lewis, S E Bohndiek

**Affiliations:** 1 Department of Physics, University of Cambridge, JJ Thomson Avenue, Cambridge CB3 0HE, United Kingdom; 2 Cancer Research UK Cambridge Institute,University of Cambridge, Robinson Way, Cambridge CB2 0RE, United Kingdom; 3 Department of Oncology, University of Oxford, Roosevelt Drive, Oxford OX3 7DQ, United Kingdom; 4 Cancer Research UK Scotland Institute, Garscube Estate, Glasgow G61 1BD, United Kingdom; 5 School of Cancer Sciences,University of Glasgow, Switchback Road, Glasgow G61 1BD, United Kingdom

**Keywords:** photoacoustic imaging, image co-registration, oncology, vasculature, computational methodologies, medical image analysis, deep learning

## Abstract

*Objective*. The formation of functional vasculature in solid tumours enables delivery of oxygen and nutrients, and is vital for effective treatment with chemotherapeutic agents. Longitudinal characterisation of vascular networks can be enabled using mesoscopic photoacoustic imaging, but requires accurate image co-registration to precisely assess local changes across disease development or in response to therapy. Co-registration in photoacoustic imaging is challenging due to the complex nature of the generated signal, including the sparsity of data, artefacts related to the illumination/detection geometry, scan-to-scan technical variability, and biological variability, such as transient changes in perfusion. To better inform the choice of co-registration algorithms, we compared five open-source methods, in physiological and pathological tissues, with the aim of aligning evolving vascular networks in tumours imaged over growth at different time-points. *Approach*. Co-registration techniques were applied to 3D vascular images acquired with photoacoustic mesoscopy from murine ears and breast cancer patient-derived xenografts, at a fixed time-point and longitudinally. Images were pre-processed and segmented using an unsupervised generative adversarial network. To compare co-registration quality in different settings, pairs of fixed and moving intensity images and/or segmentations were fed into five methods split into the following categories: affine intensity-based using (1) mutual information (MI) or (2) normalised cross-correlation (NCC) as optimisation metrics, affine shape-based using (3) NCC applied to distance-transformed segmentations or (4) iterative closest point algorithm, and deformable weakly supervised deep learning-based using (5) LocalNet co-registration. Percent-changes in Dice coefficients, surface distances, MI, structural similarity index measure and target registration errors were evaluated. *Main results*. Co-registration using MI or NCC provided similar alignment performance, better than shape-based methods. LocalNet provided accurate co-registration of substructures by optimising subfield deformation throughout the volumes, outperforming other methods, especially in the longitudinal breast cancer xenograft dataset by minimising target registration errors. *Significance*. We showed the feasibility of co-registering repeatedly or longitudinally imaged vascular networks in photoacoustic mesoscopy, taking a step towards longitudinal quantitative characterisation of these complex structures. These tools open new outlooks for monitoring tumour angiogenesis at the meso-scale and for quantifying treatment-induced co-localised alterations in the vasculature.

## 1. Introduction

Alterations in tissue vascular networks can be early indicators of a range of diseases, from psoriasis to cancer (Brown *et al* 2019, Dean-Ben and Razansky 2021). Angiogenesis in solid tumours is a hallmark of cancer, required to deliver oxygen and nutrients to the tumour mass. The evolved tumour vasculature is exploited during treatment, to deliver drug-based therapies (Lugano *et al* 2020). Thus, longitudinal imaging of the tumour vascular phenotype has become increasingly important in cancer research, both to understand how vascular biology impacts tumour evolution, but also in the context of animal welfare, considering the 3Rs (Brown *et al* 2019, Rinwa *et al* 2024). Typically, x-ray or magnetic resonance imaging modalities have been used in these studies, achieving spatial resolution of ∼0.1 mm, but rely on contrast agents to capture 3D images of the perfused vasculature (Ungersma *et al* 2010, Kersemans *et al* 2015). Higher resolution (${\sim}10$ µm) has been demonstrated with ultrasound localisation microscopy, which also relies on time-resolved tracking of contrast agents (Opacic *et al* 2018).

More recently, mesoscopic photoacoustic imaging (PAI) has emerged as an effective tool for vascular monitoring (Omar *et al* 2019, Hindelang *et al* 2022, Lefebvre *et al* 2022). Mesoscopic PAI typically excites the tissue with a 532 nm pulsed laser and enables direct non-invasive visualisation of vascular networks at high spatial resolution (∼10 *µ*m) up to ∼4 mm deep in tissue (Omar *et al* 2019). By exploiting the absorption of light by haemoglobin in red blood cells and the subsequent thermoelastic generation of acoustic waves, mesoscopic PAI produces 3D images by reconstructing the optically-induced pressure waves measured with a broadband, raster-scanned ultrasound transducer with 50MHz centre frequency coupled to the skin surface. As such, mesoscopic PAI resolves the perfused vasculature using intrinsic endogenous contrast of haemoglobin, removing the need for contrast agents. Establishing quantitative PAI biomarkers of disease-related vascular changes shows translational promise (Haedicke *et al* 2020, Hindelang *et al* 2022, Orlova *et al* 2022), but our ability to monitor and co-localise pathological changes over time is still limited by the difficulty of co-registering PAI data from different time-points under evolving conditions. Aligning mono-modal images of vascular structures can enable the precise identification of individual vessels and quantification of the changes invoked during angiogenesis (Demené *et al* 2019) or by chemo- or radiotherapy.

The image co-registration task involves spatially aligning a ‘moving’ image with another previously acquired image, referred to as the ‘fixed’ image (Hill *et al* 2001). A geometric transformation is calculated using an iterative optimisation process based on a minimisation metric to co-register the moving image to the fixed image. Two prior studies have investigated the feasibility of aligning macroscopic PAI tomography data (∼0.1–1 mm resolution): one in 2D in a phantom and healthy human hand dataset acquired with an in-house PAI system (Yu *et al* 2022), and a second on repeatedly acquired 3D breast images from one volunteer using PAI mammoscopy (Santi *et al* 2024). Aligning 3D mesoscopic PAI has not been attempted and presents particular challenges due to: (1) the sparsity of signal from vascular structures compared to the noisy background from surrounding tissue that composes most of the imaged volume; (2) image artefacts arising from the illumination geometry, limited view detection, and depth-dependent light fluence, including shadow and reflection artefacts; (3) varying signal intensities from scan to scan; and (4) operator dependence of data acquisition (Schwarz *et al* 2015, Hacker *et al* 2023).

In addition to these challenges in the data structure, there are also computational and biological challenges that impair our ability to co-register mesoscopic PAI data. Performance metrics often assess intensity similarity between images or overlap in segmented structures, however, intensity-based approaches may be limited when signal intensities are inconsistent between scans of a same anatomy such as in mesoscopic PAI, or when segmentations do not capture the full extent of the imaged structure from scan-to-scan. Furthermore, for assessing changes in vascular structures over time, biological variability, such as vasodilatory or vasoconstrictive events, vessel collapse, or changes in perfusion leading to appearance or disappearance of vessels also need to be considered since they all impact the vasculature visualised on PAI mesoscopy even on sequential scans acquired at a given time-point (Sheppard *et al* 2011, Berezhnoi *et al* 2018). Co-registration of pathological tumour vasculature adds further complexity due to abnormal vessel structures and high vessel permeability (Bergers and Benjamin 2003).

To tackle these limitations and leverage the distinctive imaging capabilities of PAI, we undertook an end-to-end testing of curated open-source co-registration methods for the alignment of 3D mesoscopic PAI data from physiological and pathological vascular structures. The main aim of the study was to enable the alignment of evolving vascular networks in tumours imaged over growth at different time-points, through the comparison co-registration techniques and performance metrics. Co-registration frameworks were tested on pre-processed intensity images and segmentation masks of vascular networks derived from an unsupervised generative adversarial network, and further compared against one another. The tested methods were evaluated on repeated scans of: (1) a healthy murine ear vasculature dataset scanned at a fixed time-point; (2) breast cancer patient-derived xenograft (PDX) tumours scanned at a fixed time-point; and (3) PDX tumours imaged longitudinally over tumour growth, representing a pathological dataset acquired at different time-points. The fixed time-point datasets were used to benchmark co-registration algorithms on repeatedly imaged vascular networks in which minimal biological variability was expected in order to assess the impact of technical variability in mesoscopic PAI on tested methods. Then, algorithms were tested on longitudinally imaged vascular networks in growing PDX where alterations in the vasculature were observed. Performance evaluation was undertaken with a number of state-of-the-art co-registration performance metrics, to assess the value of each method and metric in capturing evolving disease phenotypes using photoacoustic mesoscopy. By providing a comprehensive comparison of co-registration strategies, we are taking a first step towards quantitative longitudinal monitoring of the perfused vasculature in pathological conditions at the meso-scale, opening new outlooks in co-localised monitoring of disease progression and therapeutic response under the lens of mesoscopic PAI.

## 2. Materials and methods

### 2.1. Animal procedures

All *in vivo* procedures on small animals were conducted under project (PE12C2B96) and personal (A70F0365 and I544913B4) licenses issued by the Home Office under the Animals (Scientific Procedures) Act, 1986. Scientific procedures were carried out after review and approval from local Cancer Research UK Cambridge Institute animal welfare and ethical review bodies (compliance form numbers, SB2112, SB1567 and SB174), following latest guidance on animal welfare during the conduct of the study and for reporting findings (Kilkenny *et al* 2010, Rinwa *et al* 2024). All mice were housed in individually ventilated cages (GM500 Mouse IVC Green Line, Tecniplast, UK) in the animal facility with APB6 bedding on a 12 h on/off light/dark cycle with 5R58 diet (PicoLab) after a 7 d acclimatisation period. A total of 17 mice were used as part of this study (full details are reported in Supplementary Table 1).

Healthy female BALB/C nude mice were used for ear vasculature scans (Charles River, *n* = 5). PDX of breast cancer of luminal B subtypes (AB580 or STG139) were provided in-kind from the laboratory of Carlos Caldas at the Cancer Research UK Cambridge Institute. Human tissue used in this study were obtained after approval by the National Research Ethics Service, Cambridgeshire 2 REC (REC Reference Number: 08/H0308/178) from consenting breast cancer patients. Implantations were performed according to standard protocols (Bruna *et al* 2016, Brown *et al* 2022). Cryopreserved tumour fragments of approximately 2 mm^3^ in volume were defrosted at 37 ^∘^C, washed with media (Gibco 41 966), and mixed with matrigel (Corning 354 262) before surgical implantation into the flank of 6–9 week-old female NOD SCID gamma mice (Charles River, *n* = 12). Tumour growth was monitored with calliper measurements until tumours reached a mean diameter of 0.8 cm and were enrolled for imaging. Mice were checked everyday by animal technicians to monitor welfare and note any sign of adverse effects that would be above the license’s severity limit. Animals were culled once the average tumour diameter reached ∼1 cm.

### 2.2. *In vivo* imaging

Mesoscopic PAI of mouse ears and tumours was conducted with a raster-scan photoacoustic mesoscopy system (RSOM Explorer P50, iThera Medical GmbH) (Omar *et al* 2013). Briefly, photoacoustic signals were generated by pulsing 1 ns laser light at 1 kHz using a 532 nm wavelength at 81% laser energy guided with two optical fibre bundles, never exceeding American National Standards Institute (ANSI) safety limits. Ultrasound signals generated from tissue thermoelastic expansion were detected with a raster-scanning single-element transducer with a centre frequency of 50 MHz (${\approx}90\%$ bandwidth) attached to a motorised stage. Commercial ultrasound-gel (Aquasonic Clear, Parker Laboratories, Fairfield, NJ, USA) was used to couple the detection element with the tissue of interest after centrifugation for removal of air bubbles. The raster-scanning field of view was $12 \times 12$ mm^2^ with step size of 20 *µ*m for all images (acquisition time, ∼7 min). Prior to imaging, tumour-bearing mice were shaved in the imaging region and commercial hair removal cream was further applied to optimally couple the raster-scanning head with the mouse skin. All animals were anaesthetised using 3%–5% isoflurane administered in 50% oxygen and 50% medical air. When breathing was stabilised to 60–70 breaths per minute, anaesthesia concentration was decreased to 1%–2% isoflurane to maintain a regular breathing pattern, and therefore avoid sudden abdominal movements impacting the imaging process. Animals were then positioned on the heated bed of the mesoscopic PAI system, keeping body temperature between 36 ^∘^C–37 ^∘^C.

Healthy ears (*n* = 5) and PDX tumours located on the flank of each tumour-bearing mouse (*n* = 7) were scanned, repositioned, and scanned again from 2 to 7 times on the same day by two experienced users (LH and ELB). The first scan was systematically used as the fixed image in the co-registration workflow, and images acquired subsequently—after lifting the transducer head, manually moving the animal while preserving the tumour-transducer plane orientation, reapplying ultrasound gel if necessary, and repositioning the transducer—were defined as moving images. A total of 48 image pairs at a fixed time-point were included in this study (21 pairs of ear scans and 27 pairs of PDX scans). Longitudinal imaging was also performed in evolving PDX tumours (*n* = 5) up to 8 d apart between the fixed scan and the moving scan; 10 images were included in the longitudinal dataset (consisting of 5 image pairs). All image pairs co-registered using each proposed technique in the fixed-timepoint or the longitudinal dataset were compared against each other. Data curation was conducted retrospectively and PAI scans from the two previously acquired datasets were included (murine ears and PDX tumours).

### 2.3. Image pre-processing, post-processing, and analysis

Imaging data was reconstructed and pre-processed as described previously (Brown *et al* 2022). PAI data were reconstructed with motion correction using a previously described beam-forming algorithm to generate 3D voxelated images (Park *et al* 2008, Omar *et al* 2014). Reconstructions were performed up to a depth of 3 mm, with axial resolution of 4 *µ*m, resulting in volumetric images of $12 \times 12 \times 3$ mm^3^ with $20 \times 20 \times 4$ µm^3^ voxel size ($X \times Y \times Z$). Pre-processing involved using a high-pass filter to remove echo noise, a Wiener filter using a 3-by-3 neighbourhood for calculating pixel-wise local means and variances used in the filter to remove stochastic noise, and a slice-wise rolling ball background removal algorithm with a 5 pixel radius to homogenise background signal intensities (Brown *et al* 2022). Slice-wise *z*-score normalisation was further performed after rearranging intensity range to 0.05–99.95 percentiles, to ensure comparable signal across imaging datasets and to limit the impact of depth-dependent signal decay. This additional normalisation step was applied considering the known light fluence decrease at depth in mesoscopic PAI in order to give the same weight to imaged vessels across depths in intensity-based co-registration algorithms. Visualisations of the impact of preprocessing are provided in Supplementary figure 1.

The unsupervised image-to-image translation deep learning model, *vessel segmentation generative adversarial network* (VAN-GAN), was used for vascular network segmentation as introduced elsewhere (Sweeney *et al* 2024). VAN-GAN was employed to achieve segmentation without the need for human input, thus reducing the effect of human bias and inconsistency of manual annotations. Briefly, VAN-GAN was trained on 3D image patches obtained from photoacoustic-simulated synthetic vascular trees and their paired binary masks as ground truth (*n* = 449), and on a range of mesoscopic PAI scans of physiological and pathological vascular networks (*n* = 729), which were randomly rotated about the *Z*-axis for data augmentation. Prior to training and segmentation, the only preprocessing steps applied were the aforementioned slice-wise *z*-score normalisation and thresholding of intensity range, and then intensities were re-arranged between −1 and 1. The full dataset was split in the following way: 90% assigned to training and validation (80% training/20% validation) and the remaining 10% for testing. VAN-GAN was adapted from the cycle GAN architecture to learn the mapping between the imaging and segmentation domain using generator and discriminator blocks with tailored loss functions during training over the course of 200 epochs using the Adam optimiser (Kingma and Ba 2014). Further details are provided in the original publication and in the openly available code deposited on an online repository (https://github.com/psweens/VAN-GAN) (Sweeney *et al* 2024). VAN-GAN segmentations were obtained for all ear and PDX mesoscopic PAI scans, after resampling data to isotropic resolution; these segmentations were used independently for testing shape-based co-registration.

Finally, in the ear dataset, regions of interest (ROI) including only reconstructed *Z*-slices with vessel signals were selected in the moving images to reduce the impact of reflection and shadow artefacts on the co-registration process. In the tumour dataset, a pre-trained 3D convolutional neural network based on the U-Net architecture described elsewhere (Brown *et al* 2022) was used to delineate tumour regions to produce ROIs, in order to limit the inclusion of background signal in the co-registration process.

### 2.4. Intensity-based image co-registration

**Figure 1.**
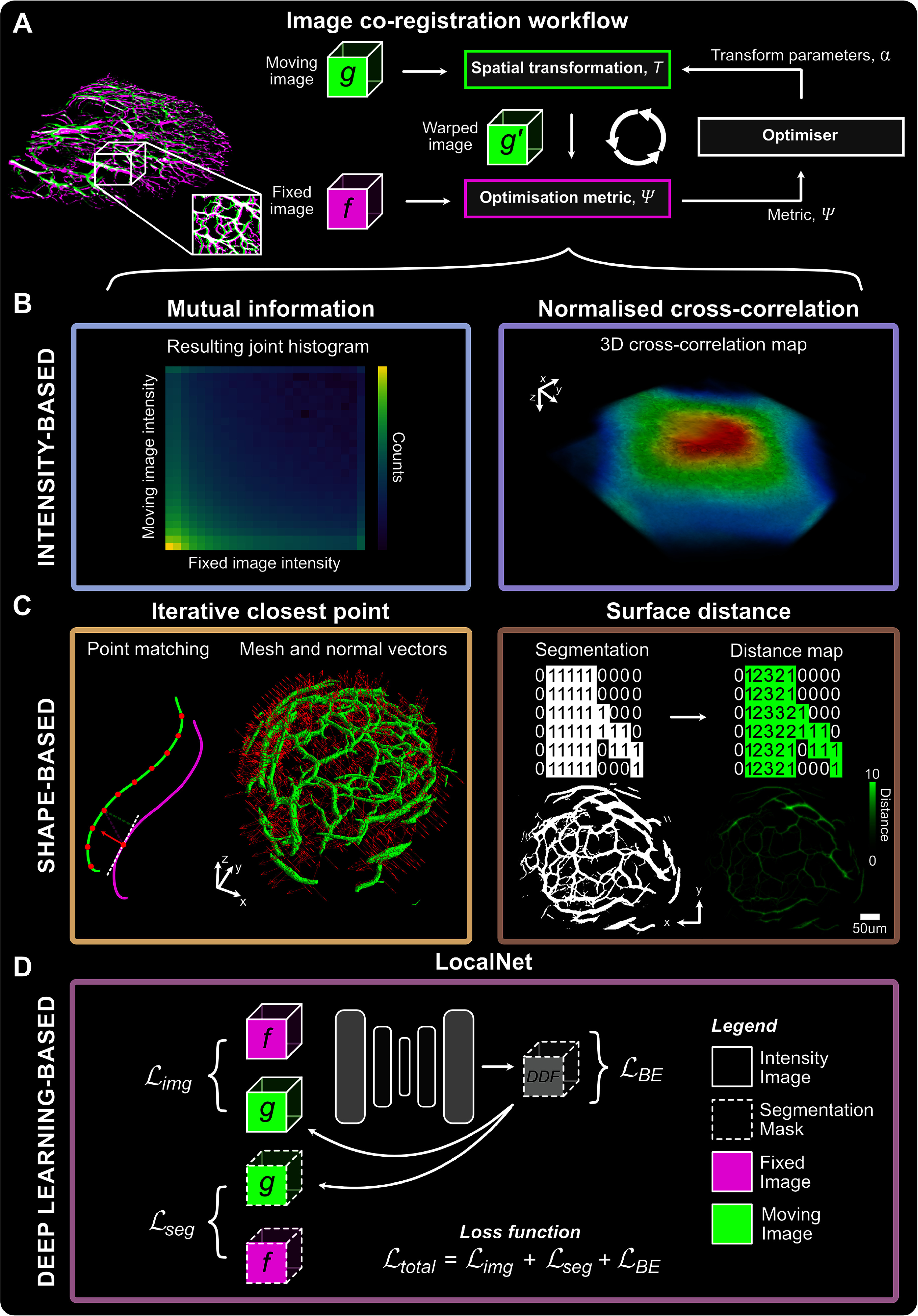
Curated computational methods for the co-registration of longitudinal 3D vascular photoacoustic mesoscopy. (A) Overview of the general image co-registration workflow in which spatial transform parameters are calculated to minimise a pre-selected metric iteratively, with the goal of maximising the similarity or overlap of imaged vascular structures. (B) Intensity-based methods align pre-processed images based either on mutual information (MI) or normalised cross-correlation (NCC). (C) Shape-based co-registration methods use either the iterative closest point (ICP) algorithm on point cloud-transformed segmentations, iteratively matching the moving point cloud to the fixed one while minimising a mean squared error function, or distance-transformed segmentations using NCC as an optimisation metric. (D) Deep learning-based co-registration, LocalNet, takes as an input paired intensity images and segmentations to assess the deformation field minimising intensity-based, segmentation-based, and bending energy losses during weakly supervised training. BE, bending energy; DDF, dense displacement field.

The proposed image co-registration workflows (figure 1(A)) were implemented using readily-available open-source Python packages. All the code used in this study is openly available online (https://github.com/BohndiekLab/ coregistration-longitudinal-pai-mesoscopy). The multi-dimensional image analysis Python package, simple insight toolkit (SimpleITK, v2.2.1), was used for traditional intensity-based co-registration of photoacoustic mesoscopic images (figure 1(B)) (Lowekamp *et al* 2013). Mutual information (MI) or NCC were used as optimisation metrics. After an initial rigid alignment based on centres of mass of both images, moving images were iteratively spatially transformed to optimise intensity-based metrics (NCC or MI) using gradient descent until a 100-iteration threshold was reached, or when a minimum was found. The same optimisation procedure was applied on distance-transformed images described in the following section.

Given two image volumes, the fixed image $f(\vec{r})$ shown in magenta in all figures, and the moving image $g(\vec{r})$ shown in bright green (where $\vec{r} = (x,y,z)$), a 3D spatial transformation, $\vec{T} : \mathbb{R}^3 \to \mathbb{R}^3$, was calculated between the two image domains to optimise the similarity between the two images, based on MI or NCC. The spatially transformed or ‘warped’ image, $g(\vec{T}(\vec{r}))$ was moved iteratively based on those predefined similarity metrics, $\Psi(f(\vec{r}),g(\vec{T}(\vec{r};\boldsymbol\alpha)))$, optimising based on the parameterisation of ***α***, which encodes transformation parameters such as 3D translations and rotations.

Tested similarity metrics $\Psi(f(\vec{r}),g(\vec{T}(\vec{r})))$ included MI calculated from the binned intensity histograms of images $f(\vec{r})$ and $g(\vec{T}(\vec{r}))$, *F* and *G* respectively, as:

\begin{align*} \Psi\left(f\left(\vec{r}\right),g\left(\vec{T}\left(\vec{r}\right)\right)\right) = \text{MI}\left(F,G\right) = H\left(F\right) + H\left(G\right) - H\left(F,G\right),\end{align*}

where *H*(*F*) and *H*(*G*) are the marginal entropies, which are indicative of how much information we learn on average from one instance in the binned intensity histogram of $f(\vec{r})$, *F*, and in the intensity histogram of $g(\vec{T}(\vec{r}))$, *G*. The function $H(F,G)$ is the joint entropy which measures the uncertainty in the two intensity distributions *F* and *G*. Hence, maximising MI involves matching spatially the most complex regions of each image by maximising individual marginal entropies, such that they explain each other’s information by minimising joint entropy (Thevenaz and Unser 2000).

The other tested similarity metric in intensity-based image co-registration was NCC:

\begin{align*} \Psi\left(f\left(\vec{r}\right),g\left(\vec{T}\left(\vec{r}\right)\right)\right) = \text{NCC}\left(f\left(\vec{r}\right),g\left(\vec{T}\left(\vec{r}\right)\right)\right) = \frac{\sum_i \left(f\left(\vec{r}\right)-\bar{f\,}\,\right) \cdot \left(g\left(\vec{T}\left(\vec{r}\right)\right)-\bar{g}\right)}{\left[\sum_i \left(f\left(\vec{r}\right)-\bar{f}\,\right)^2 \cdot \sum_i \left(g\left(\vec{T}\left(\vec{r}\right)\right)-\bar{g}\right)^2\right]^{1 / 2}}.\end{align*}

NCC is mathematically expressed as the dot product of the fixed image $f(\vec{r})$, and the warped image $g(\vec{T}(\vec{r}))$, over all voxels *i*, centred around their respective means, $\bar{f}$ and $\bar{g}$, divided by the square root of the product of the $L2$-norm of each image. Thus, NCC quantifies localised image similarity based on voxel intensities.

The selected metrics $\Psi(f(\vec{r}),g(\vec{T}(\vec{r})))$ must be differentiable for gradient descent-based evaluation of the spatial transformation. The iterative gradient descent problem can be stated as the change in objective function when the parameterisation ***α*** of the transformation $\vec{T}$ changes over iterations, $n \in \{1,{\ldots},N \}$, to maximise the quality metric:

\begin{align*} \hat{\boldsymbol\alpha} = \arg\max_{\boldsymbol\alpha}\Psi\left(f\left(\vec{r}\right),g\left(\vec{T}\left(\vec{r};\boldsymbol\alpha\right)\right)\right).\end{align*}

The parameterisation of the transformation matrix is estimated by having the optimiser take iterative steps in the multi-dimensional space where the optimisation metric is maximised against those individual transformation parameters in the direction of the gradient of that metric:

\begin{align*} \hat{\boldsymbol\alpha}_{n+1} = \hat{\boldsymbol\alpha}_n - \gamma \nabla_{\boldsymbol\alpha} \Psi\left(f\left(\vec{r}\right),g\left(\vec{T}\left(\vec{r};\boldsymbol\alpha\right)\right)\right),\end{align*}

where

\begin{align*} \nabla_{\boldsymbol\alpha} \Psi = \left(\begin{array}{c} \frac{\partial \Psi}{\partial \alpha_1} \\ \vdots \\ \frac{\partial \Psi}{\partial \alpha_k} \end{array}\right).\end{align*}

Here, $\partial \Psi/\partial \alpha_k$ is the partial derivative of the optimisation metric against each parameter of the transformation matrix, *α*_*k*_, and *γ* is the preset learning rate. The transformation is then calculated, applied to the moving image, the optimisation metric between the warped and fixed images is updated, and the next iteration takes place (figure 1). The iterative co-registration process is ended if the maximum number of iterations of 100 was reached or if the current iteration minimum convergence value in the gradient descent optimiser is smaller than $1 \times 10^{-8}$. The warped moving and fixed image pairs were then displayed by overlaying their maximum intensity projections (MIP) taken along the *Z* axis, where green was systematically used to represent mismatched signals from the moving image, magenta represented mismatched signals from the fixed image, and where white represented the matched overlapping signals.

In the longitudinal PDX dataset, where the most substantial changes in the vasculature were observed between paired images, the binary VAN-GAN segmentation masks were dilated slice-wise along the *Z* axis using a flat disk-shaped footprint and applied to the intensity images to limit the inclusion of background signal for intensity-based co-registration using the open-source scikit-image Python package (skimage, v0.21.0) (van der Walt *et al* 2014). Moreover, instead of initialising the co-registration with centre-of-mass alignment, three landmarks were manually selected by an experienced user (TLL) in the paired images corresponding to the same visually identified branching vessels, and the co-registration process was initialised by conducting rigid 3D alignment of those landmarks, prior to the iterative co-registration of the selected pairs.

### 2.5. Shape-based image co-registration

Shape-based affine co-registration was evaluated using the point-to-plane iterative closest point (ICP) algorithm, or using surface distance-transformed segmentations for co-registration with NCC as an optimisation metric (figure 1(C)).

ICP and its derivatives have been a mainstay in 3D shapes registration for computer vision tasks but its application has been limited in biomedical imaging of the vasculature, with few applications in brain (Reinertsen *et al* 2007) or retinal angiography (Stewart *et al* 2003). ICP was performed using the Open3D Python package (open3D, v0.17) (Chen and Medioni 1992, Zhou *et al* 2018). 3D voxelated segmented data were converted to surface meshes using a topology preserving marching cubes algorithm with one step per voxel (Lewiner *et al* 2003). The surface vertices were loaded as a point cloud with their surface normals. Point-to-plane ICP was performed on resulting fixed and moving surface point clouds, $F_s(\vec{r}) = \{\,f_{s,1}, {\ldots}, f_{s,N} \}$ and $G_s(\vec{r}) = \{g_{s,1}, {\ldots}, g_{s,M} \}$ (figure 1(C)). In ICP, moving and fixed segmentations were transformed into point clouds and points from the moving cloud were iteratively associated to the fixed cloud based on nearest neighbours minimising a mean square error cost function. Over each iteration, a set of neighboring points are found and paired, for correspondence $\kappa = \{(f_{s,1},g_{s,1}), {\ldots}, (f_{s,N},g_{s,M}) \}$. The cost function for the point cloud is given by the sum of the squared distance between each source point and the tangent plane at its corresponding destination point, along the surface normals *η*:

\begin{align*} \Psi\left(F_s\left(\vec{r}\right),G_s\left(\vec{T}\left(\vec{r}\right)\right)\right) = \sum_i \lVert \left[ g_{s,i}\left(\vec{T}\left(\vec{r}\right)\right)-f_{s,i}\left(\vec{r}\right) \right] \cdot \eta_i \rVert_2 .\end{align*}

The parameters of the $4 \times 4$ affine transform matrix are optimised iteratively as the closest points pairs *κ* are iteratively matched up to a 1000 iterations as per equation (4).

For surface distance-based co-registration, binary segmentations obtained with VAN-GAN were converted into Euclidean distance maps using the Maurer algorithm (Maurer *et al* 2003) with the SciPy multidimensional image processing package (scipy.ndimage, v1.9.1). Each voxel in the output 3D images takes an integer value corresponding to the distance in voxels from the closest surrounding background voxel (figure 1(C)). This adds weighting to the central line along segmented vessel where higher values are obtained as the central line stands the furthest from the background. The moving images are aligned to the fixed images in the same process as described above for the intensity-based co-registration methods, using NCC as an optimisation metric.

### 2.6. Deep learning-based image co-registration

Deep learning-based deformable co-registration was tested using a weakly supervised dual channel input (intensity images and segmentations) U-Net (figure 1(D)). The Medical Open Network for Artificial Intelligence (MONAI, v1.2, https://monai.io/) Python package was employed (Cardoso *et al* 2022). The LocalNet method, introduced in Hu *et al* (Hu *et al* 2018a, 2018b), is based on the U-Net architecture for ultrasound to magnetic resonance imaging co-registration of prostate images. LocalNet was trained on paired intensity images and segmentations in a weakly supervised fashion.

LocalNet was trained to learn the deformable transformation between moving intensity images and segmentations and the fixed ones (cropped to a $512 \times 512 \times 128$ voxels field of view in $X \times Y \times Z$ for computational efficiency). A four-layer depth was used in the encoder-decoder architecture with He initialisation on the last layer of the U-Net architecture (summarised in figure 1(D); architecture shown in Supplementary figure 2). The Adam optimiser was used for updating network weights during training, with a learning rate of 10^−4^ (Kingma and Ba 2014). The total loss function was composed of NCC loss as per equation (2), but negative, as an intensity-based loss ($L_\textrm{img}$), generalised Dice loss (GDL) as a segmentation-based loss ($L_\textrm{seg}$) (Sudre *et al* 2017), and bending energy (BE)-based regularisation of the output deformation fields ($L_\textrm{BE}$) (Rueckert *et al* 1999), and was minimised and backpropagated over the course of 1000 epochs. GDL is defined mathematically as the intersection of segmented voxels between moving and fixed segmentations over their union but adding weighting to each label *l* (foreground vs background) to account for their imbalanced distribution:

\begin{align*} \mathrm{GDL} = 1-2 \frac{\sum_{l = 1}^2 \left[\left(1 /\sum_{i} f_{l, i}\right)^2 \sum_i f_{l, i} g_{l, i}\right]}{\sum_{l = 1}^2 \left[\left(1 /\sum_{i} f_{l, i}\right)^2 \sum_i \left(f_{l, i}+g_{l, i}\right)\right]}.\end{align*}

$L_\textrm{BE}$ is defined as the sum over all voxels of the squared Hessian of the output deformable co-registration field, **T**, ensuring smoothness of the local deformations by minimising their second derivatives, and hence resulting in a locally affine transformation:

\begin{align*} L_\textrm{BE} = \frac{1}{V} \sum_{\vec{r} \in \mathbb{R}^3}\left\|\nabla^2 \mathbf{T}\left(\vec{r}\right)\right\|^2.\end{align*}

Thus, the resulting loss function was defined as the sum of each individual loss:

\begin{align*} L_\textrm{total} = L_\textrm{img}+L_\textrm{seg}+L_\textrm{BE}.\end{align*}

Losses over epochs during training along with generalised Dice in the validation dataset are shown in Supplementary figure 3. The term weakly supervised is used since the network learns over epochs to align 3D volumes without the need for ground truth. A 50/50-split of the fixed time-point ear and PDX datasets was used for training/validation (*n* = 24/24 image pairs) and loaded in batch size of 1. Small rotations about the *Z*-axis were applied for data augmentation during training (up to $\pi/15$ rad). The trained network was further applied on image pairs from the longitudinal dataset in PDXs for additional testing on an unseen dataset (*n* = 5 image pairs).

### 2.7. Quality metrics

Paired intensity images and binary segmentation masks were used to calculate quality metrics for the co-registration process. The metrics for this task were selected based on the recommendations of Maier–Hein *et al* (Maier-Hein and Menze 2022). Percent differences in quality metrics pre- vs post-registration are reported for direct comparison between methods. Delta quality metrics were obtained for segmentation-based metrics: Dice coefficients, Hausdorff distance (HD) and average Maurer distance (MD), and intensity-based metrics: MI and structural similarity index metric (SSIM). Dice coefficients are defined as the intersection of segmented voxels between the moving segmentation *G* and fixed segmentation *F* masks over the union of voxels in both images (Dice 1945):

\begin{align*} \text {Dice } = \frac{2|G \cap F|}{|G|+|F|}.\end{align*}

The surface distance $d\left(\vec{r}_{G_s}, F_s\right)$ was defined by considering the surface of the moving mask *G_s_* and surface of the fixed mask *F_s_*:

\begin{align*} d\left(\vec{r}_{G_s}, F_s\right) = \min _{\vec{r}_{F_s} \in F_s} d\left(\vec{r}_{G_s}, \vec{r}_{F_s}\right),\end{align*}

where $\vec{r}_{G_s}$ and $\vec{r}_{F_s}$ are points on the propagated surface *G_s_* and reference surface *F_s_*, was used to calculate the average surface distance, or MD:

\begin{align*} \text{MD} = \frac{1}{\left|G_s\right|+\left|F_s\right|}\left(\sum_{\vec{r}_{G_s} \in G_s} d\left(\vec{r}_{G_s}, F_s\right)+\sum_{\vec{r}_{F_s} \in F_s} d\left(\vec{r}_{F_s}, G_s\right)\right).\end{align*}

We calculated the symmetric HD as:

\begin{align*} \text{HD} = \max \left\{d_H\left(G_s, F_s\right), d_H\left(F_s, G_s\right)\right\},\end{align*}

where

\begin{align*} d_H\left(G_s, F_s\right) = \max _{\vec{r}_{G_s} \in G_s} \min _{\vec{r}_{F_s} \in F_s} d\left(\vec{r}_{G_s}, \vec{r}_{F_s}\right).\end{align*}

Finally, the SSIM was defined as a function of a luminance, a contrast, and a structural term, which can be expressed as a function of local means of the fixed (*µ*_*f*_) and moving image (*µ*_*g*_), standard deviations of the fixed (*σ*_*f*_) and moving image (*σ*_*g*_), and their cross-covariance (*σ*_*fg*_) (Wang *et al* 2004):

\begin{align*} \textrm{SSIM}\left(f\left(\vec{r}\right), g\left(\vec{r}\right)\right) = \frac{\left(2 \mu_f \mu_g+C_1\right)\left(2 \sigma_{f g}+C_2\right)}{\left(\mu_f^2+\mu_g^2+C_1\right)\left(\sigma_f^2+\sigma_g^2+C_2\right)},\end{align*}

where $C_1, C_2,$ and *C*_3_ are constants expressed as a function of the dynamic range *L* of the intensity histogram of the images as $C_1 = (0.01 L)^2; C_2 = (0.03 L)^2; C_3 = C_2/2$. The computation times of all co-registration methods in the murine ear dataset were also measured and reported on a dual 8-core Intel Xeon E5-2623 v4 central processing unit at 2.60 GHz with 64 GB of random access memory, and with a 8 GB NVIDIA GeForce GTX 1070 Ti graphics processing unit.

### 2.8. Statistical analyses

Statistical analyses were conducted in Python with the SciPy statistical functions module (scipy.stats, v1.9.1) and the statsmodels package (statsmodels, v0.14.1). Descriptive statistics of the distribution of quality metrics were computed and visualised with box and whisker plots using the seaborn Python package (seaborn, v0.11.2) (Waskom 2021). Non-parametric comparisons of quality metrics obtained between each pair of co-registration methods were performed using Wilcoxon signed rank test (for continuous, non-normally distributed paired data) adjusting significance level with Holm–Bonferroni multiple comparison corrections (comparisons number, *m* = 10) (Holm 1979, Wilcoxon 1992). All adjusted *P*-values smaller than a significance level of *α* = 0.05 were considered significant. Holm’s extension of the Bonferroni multiplicity correction was employed to ensure that the risk of rejecting one or more true null hypotheses, *H*_0_, is at *α*, while better controlling the risk of failing to reject one or more false *H*_0_, in a step-down adjustment approach, going from the smallest to the largest *P*-value.

## 3. Results

### 3.1. Co-registration evaluation on repeated scans of physiological and pathological vasculature at a fixed time-point

**Figure 2.**
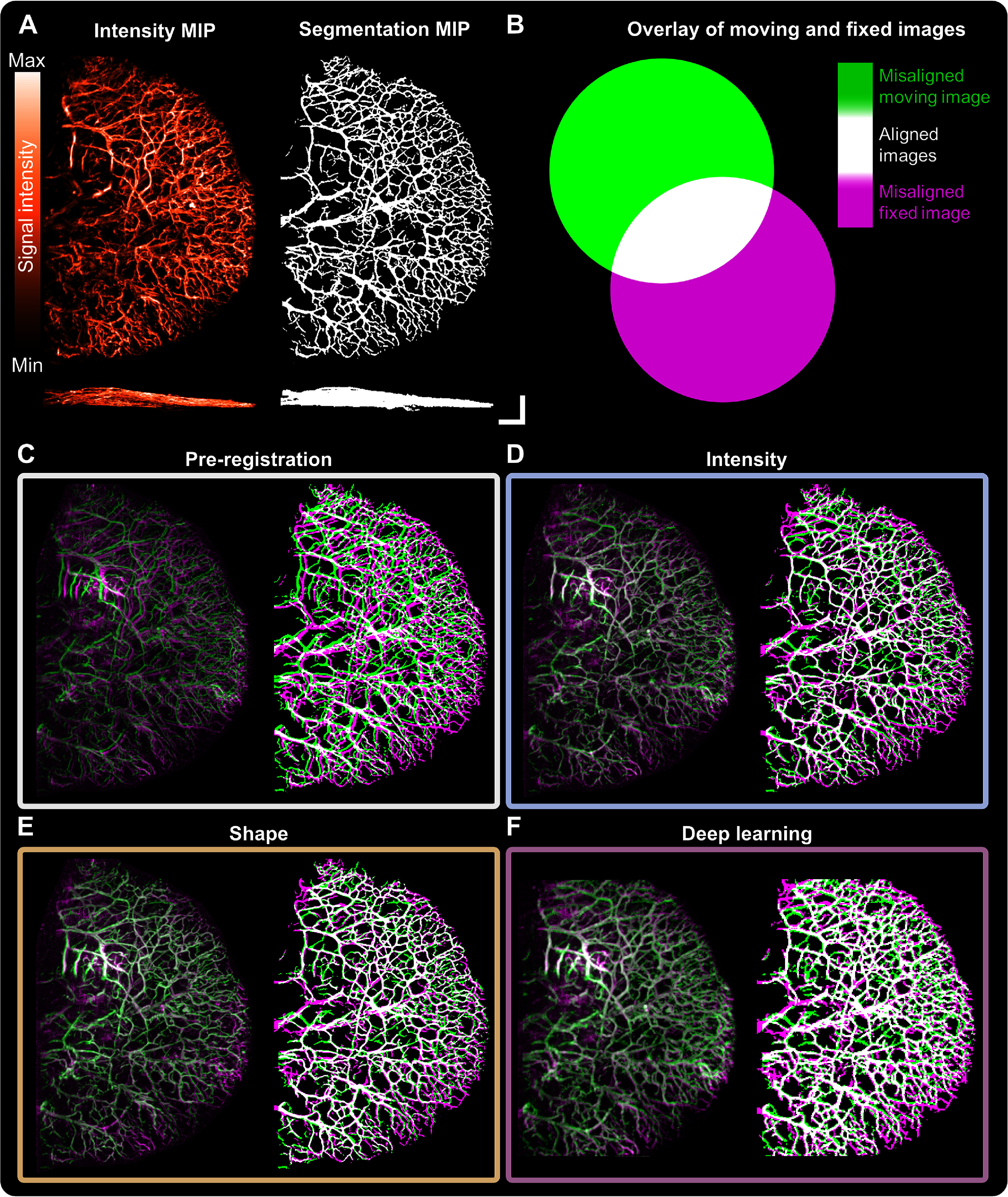
Exemplar maximal intensity projections (MIP) demonstrating mesoscopic photoacoustic image co-registration for repeated scans at a fixed time-point of healthy murine ear vasculature. (A) Fixed intensity image and segmentation (upper *XY*, lower *XZ* MIPs). (B) Moving intensity image and segmentation (upper *XY*, lower *XZ* MIPs). Scale bars, 1 mm. Intensity and segmentation MIP overlays for co-registration methodology in each category: (C) pre-co-registration, and post-co-registration using either (D) intensity-based (mutual information), (E) shape-based (iterative closest point algorithm), or (F) deep learning-based (LocalNet) methods. NB: small sections on the *Y* axis of the ear images were cropped to respect size limitations of LocalNet.

**Figure 3.**
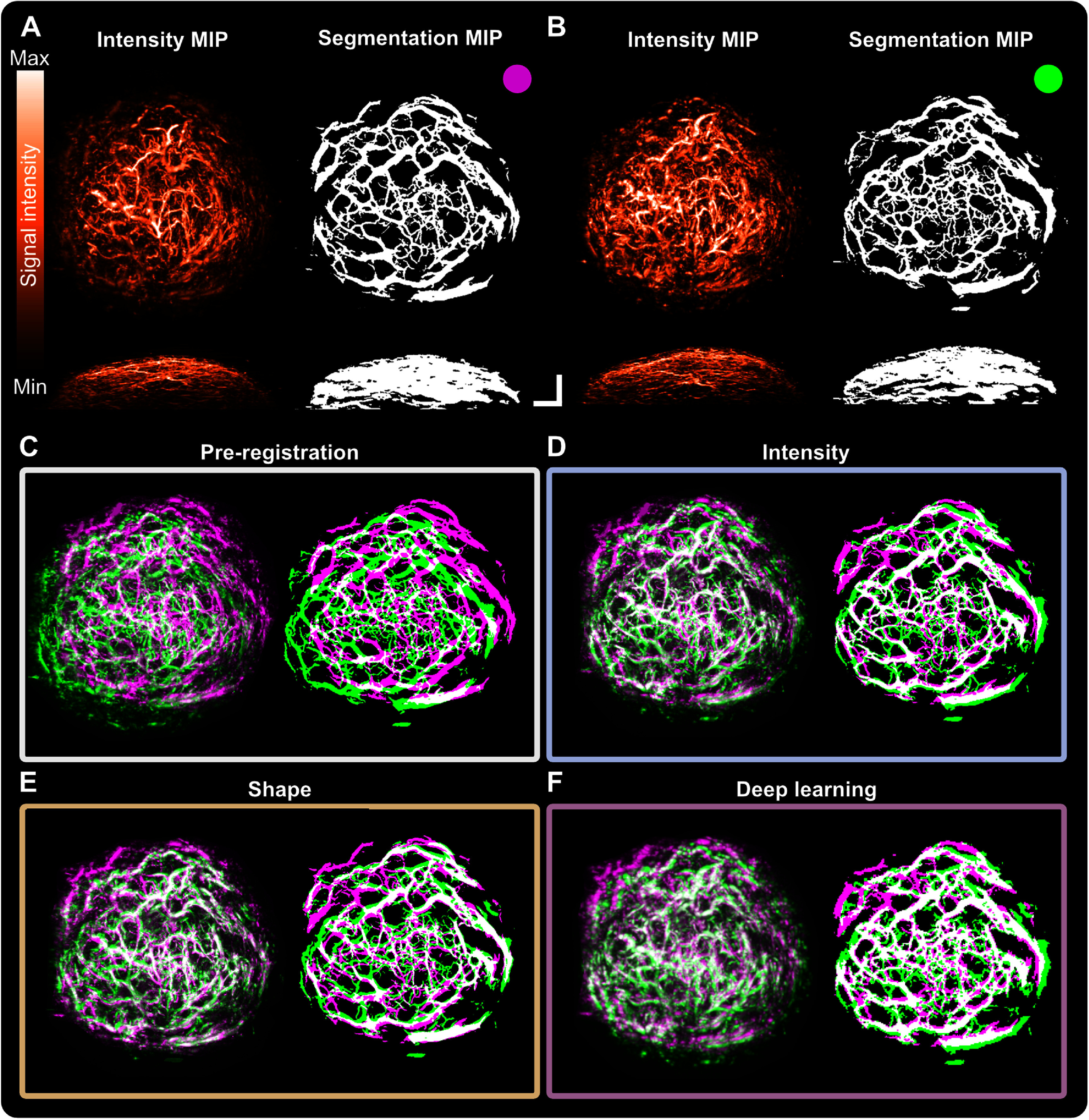
Exemplar maximal intensity projections (MIP) demonstrating mesoscopic photoacoustic image co-registration for repeated scans at a fixed time-point of breast cancer patient-derived xenografts’ vasculature. (A) Fixed intensity image and segmentation (upper *XY*, lower *XZ* MIPs). (B) Moving intensity image and segmentation (upper *XY*, lower *XZ* MIPs). Scale bars, 1 mm. Intensity and segmentation MIP overlays for each co-registration methodology: (C) pre-co-registration, and post-co-registration using either (D) intensity-based (mutual information), (E) shape-based (iterative closest point algorithm), or (F) deep learning-based (LocalNet) methods.

Co-registration methods were first applied on mesoscopic PAI pairs from repeated scans of healthy vasculature (mouse ears) or pathological vasculature (PDX tumours) acquired on the same day (figures 2 and 3, (A)–(B); Supplementary figures 4 and 5). Upon visual examination, intensity-based and shape-based methods seemed to adequately align larger vascular structures while missing smaller vessels in both fixed and warped moving images (figures 2 and 3(C)–(E)), while deep learning-based co-registration was able to evaluate required local deformations to co-register smaller vessels missed in other methods (figures 2 and 3, (F)). Additional co-registration pairs including NCC and NCC on distance-transformed images are shown in Supplementary figure 6.

**Figure 4.**
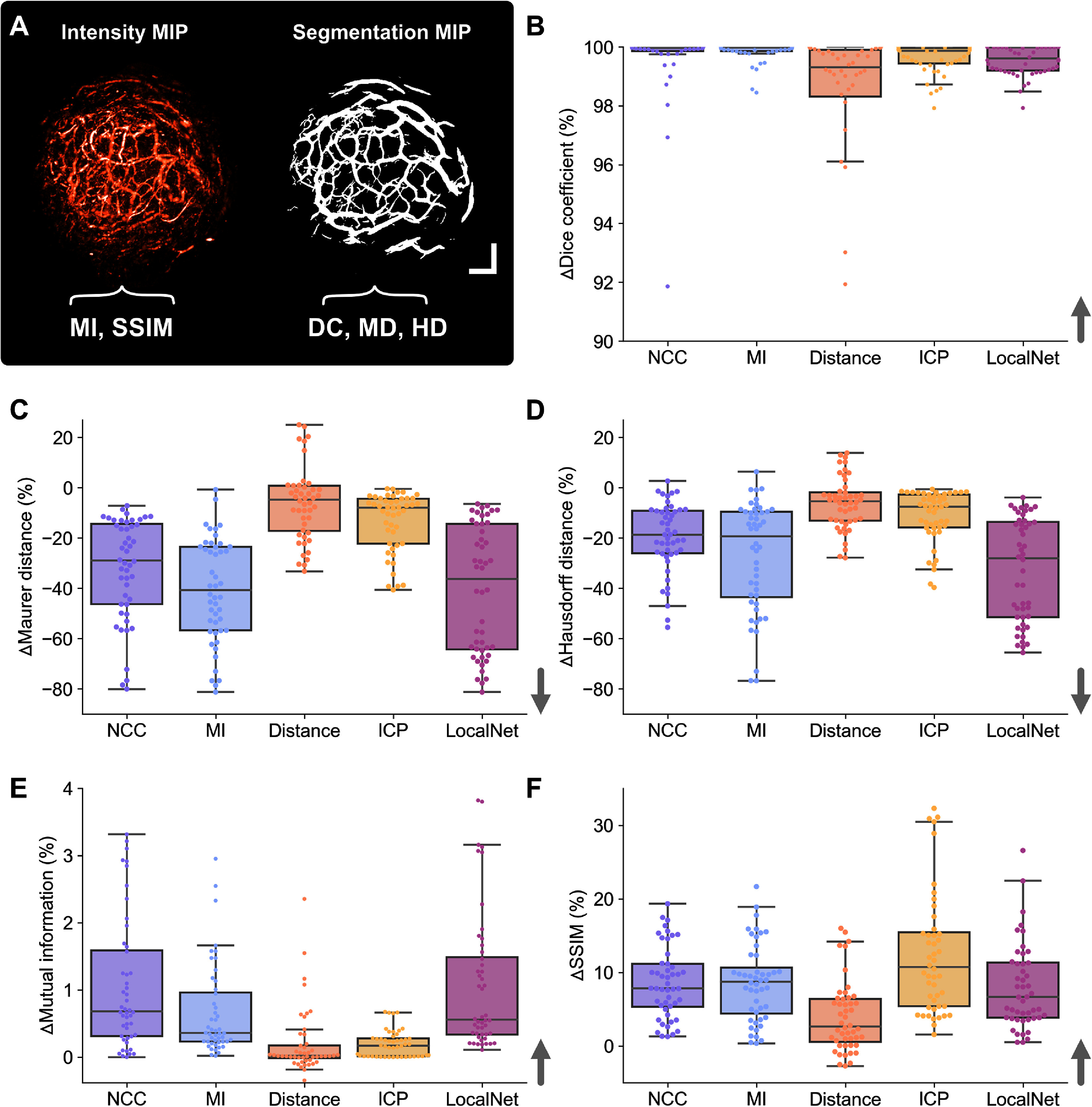
Percent change in quality metrics pre- vs post-image co-registration in the fixed time-point dataset on ear and tumour vasculature. Quantification performance is plotted for each of: intensity-based methods (labelled as NCC or MI), shape-based methods (labelled as distance or ICP), and the deep-learning method (labelled as LocalNet). Quality metrics measured on co-registered (A) intensity images or segmentations pairs include: (B) Dice coefficient, (C) Maurer distance, (D) Hausdorff distance, (E) mutual information, and (F) structural similarity index measure (SSIM). *Notes:* Arrows indicate the direction that shows improvement in co-registration performance. Scale bars, 1 mm.

The percent change in quality metrics pre- vs post-co-registration was then calculated (figure 4 and Supplementary table 2). In the fixed time-point datasets (figure 4(A)), for segmentation-based quality metrics, average ΔDice was over 97.0% for all tested methods in both datasets, except in PDX distance-transformed PAI co-registration where an average 94.40% improvement was obtained (figure 4(B), *P*$ < $0.01 for all methods). Surface distance-based metrics decreased the most in NCC, MI and LocalNet co-registered ear/tumour data with an average ΔMD (figure 4(C)) of −29.0%/−34.7%, −42.1%/−37.7%, and −66.9%/−20.2%, respectively and an average ΔHD (figure 4(D)) of −18.4%/−20.5%, −40.8%/−16.3%, and −53.9%/−16.3%, respectively. A significantly smaller decrease in surface distances was observed for ICP and distance-transformed segmentation co-registered ear/tumour data, respectively −9.1%/−16.8% and −10.5%/−0.6% in terms of ΔMDs, and −6.1%/−15.4% and −9.8%/−3.4% in terms of ΔHD (*P*$ < $0.01 for all comparisons with NCC, MI and LocalNet).

For intensity-based quality metrics, a marginal improvement was observed in ΔMI of less than 2% across tested methods (figure 4(E)). The highest improvements were observed for NCC and LocalNet in the ear/tumour datasets, with 0.48%/1.50% and 2.00%/0.36%, respectively (*P*$ < $0.001 for all comparisons except between NCC/MI/LocalNet). ΔSSIM was higher in ICP-registered and LocalNet-registered ear/PDX images with average values of 6.62%/17.58% and 9.05%/8.18%, respectively (figure 4(F)). ICP provided significantly higher ΔSSIM than all tested methods except LocalNet (*P*$ < $0.01 for all comparisons).

Overall, only MI, NCC, and LocalNet co-registration frameworks were superior (or at least non-inferior) on average to other methods based on evaluated quality metrics. ICP resulted in improved image similarity post-registration only in terms of SSIM. A further consideration when selecting a co-registration method is the required computational time (table 1). After training was completed, the selected deep learning technique was significantly faster than all methods when applied on image/segmentation pairs with an average time of $3.3 \pm 0.2$ s (*P*<0.01, compared to all methods). ICP achieved the second fastest computation time with $6.0 \pm 11.2$ s on average per co-registration, significantly lower than NCC-, MI-, and distance-based methods (*P*<0.001), and followed by NCC with $66.5 \pm 22.1$ s, which was significantly quicker than the distance-based method only (*P*<0.01).

**Table 1.** Computation time of the 3D spatial transformation in tested image co-registration frameworks on the murine ear dataset. Data presented as mean ± standard deviation (*n* = 21 image pairs).

Co-registration method	Time (s)
NCC	66.5 ± 22.1
MI	78.1 ± 75.0
Distance	122.6 ± 101.8
ICP	6.0 ± 11.2
LocalNet	3.3 ± 0.2

### 3.2. Co-registration evaluation on longitudinally imaged vasculature in a breast cancer model

**Figure 5.**
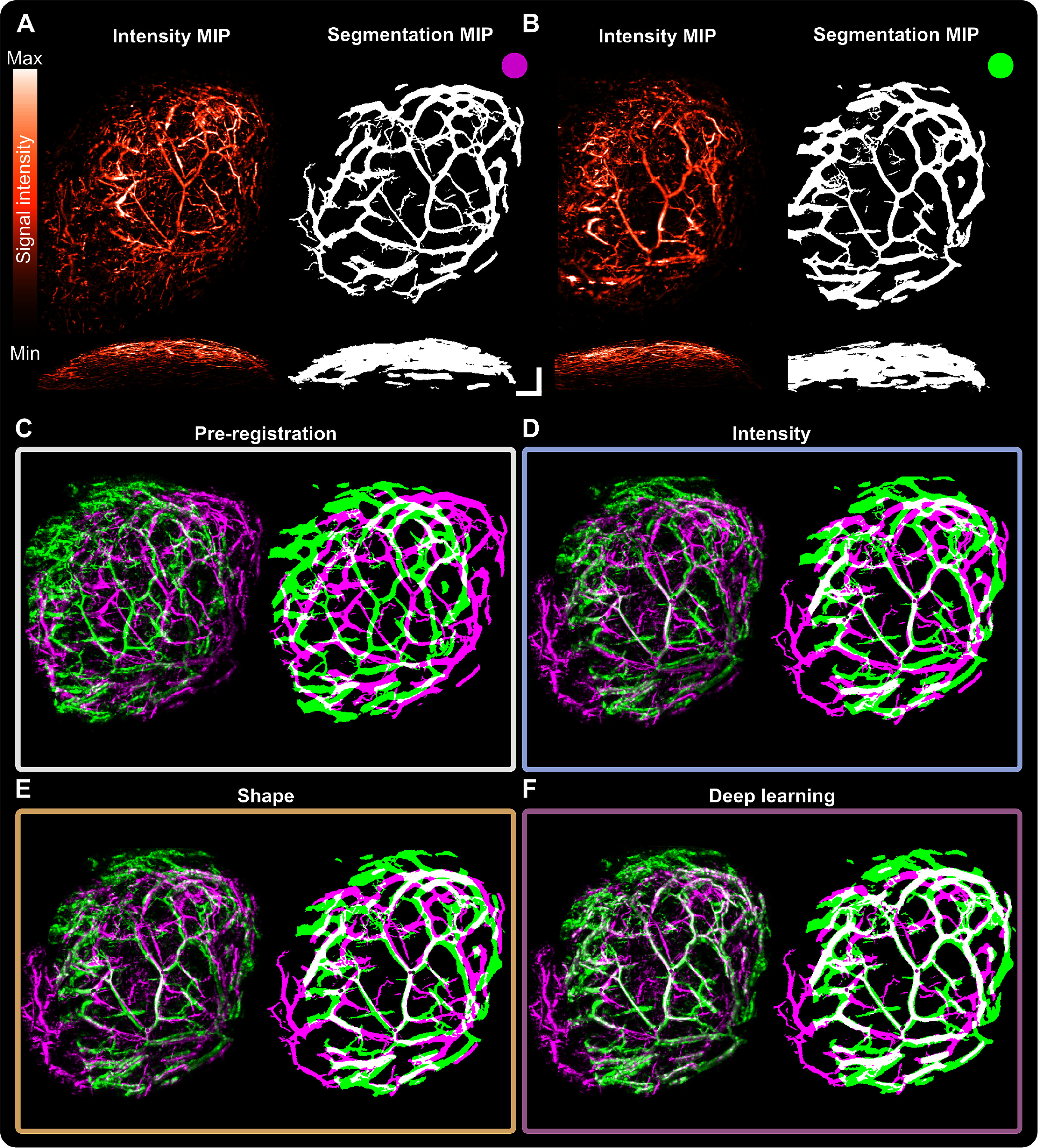
Exemplar MIPs of mesoscopic PAI co-registration for tumours acquired longitudinally with 3–8 d between scans.(A) Fixed intensity image and segmentation (upper *XY*, lower *XZ* MIPs). (B) Moving intensity image and segmentation (upper *XY*, lower *XZ* MIPs). Scale bar, 1 mm. Pre- and post-co-registration pairs for intensity images and segmentations of each co-registration method: (C) pre-co-registration, and post-co-registration using either (D) intensity-based (mutual information), (E) shape-based (iterative closest point algorithm), or (F) deep learning-based (LocalNet) methods. Manually selected landmarks are overlaid on co-registered image pairs in green and purple.

**Figure 6.**
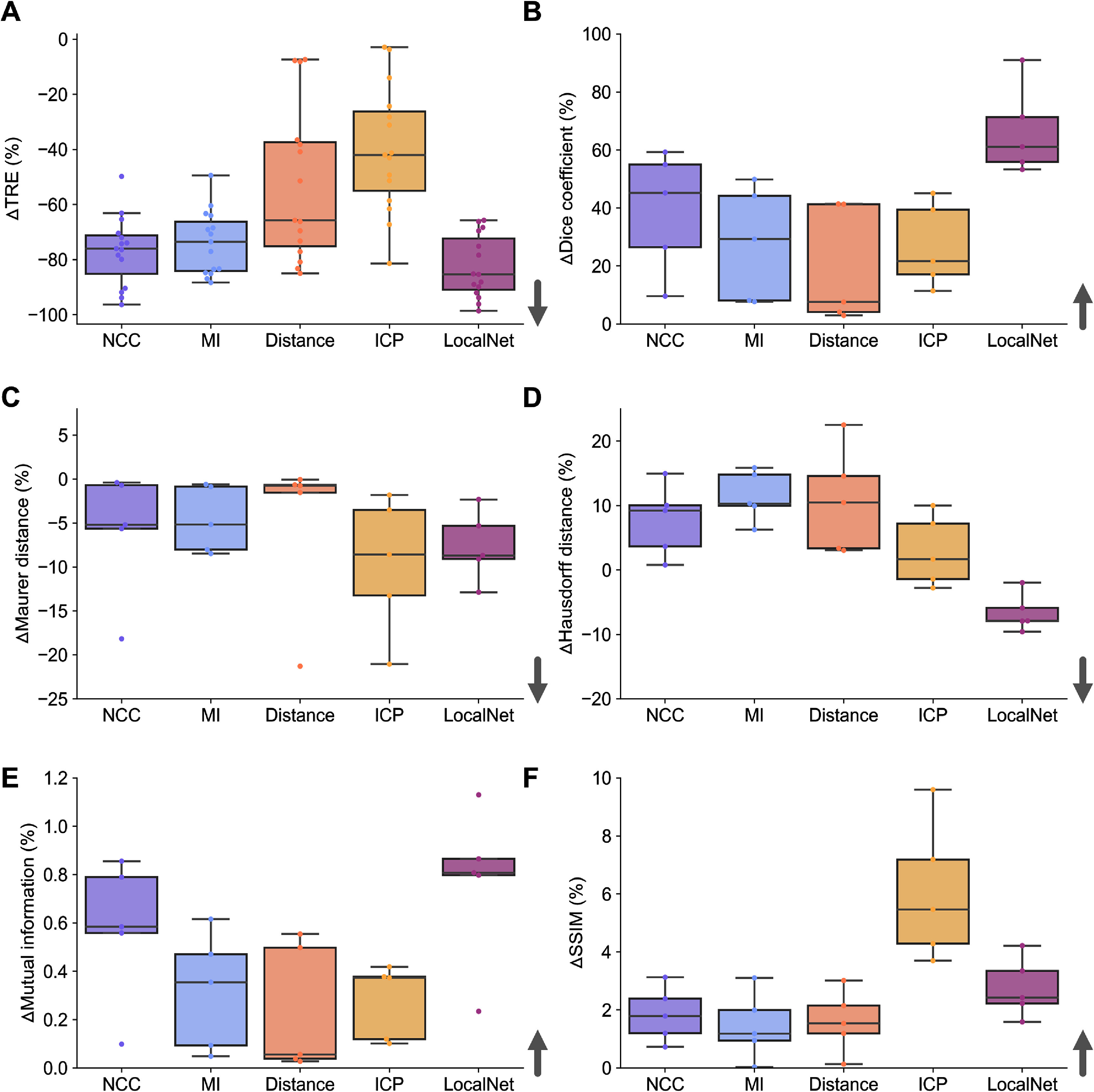
Percent change in quality metrics pre- vs post-image co-registration in the longitudinal dataset on tumour vasculature. Quantification performance is graphed for each of: intensity-based methods (labelled as NCC or MI), shape-based methods (labelled as distance or ICP), and the deep-learning method (labelled as LocalNet). For each co-registration method, improvements are reported in: (A) target registration error, (B) Dice coefficient, (C) Maurer distance, (D) Hausdorff distance, (E) mutual information, and (F) structural similarity index measure. Arrows indicate the direction of improvement.

Selected co-registration techniques were then applied on mesoscopic PAI pairs from scans of evolving breast tumour vasculature in PDXs acquired at two time-points (figure 5; exemplar landmarks demonstrated in supplementary figure 7). Percent changes in quality metrics were calculated (figure 6 and Supplementary table 3), also including target registration errors (TRE) between the 3 pairs of manually selected landmarks in 3D for each image pair (total of 15 landmarks). The number of days between scans ranged from 3 to 8 d (mean time between scans, $6 \pm 3$ d), leading to more substantial changes in the imaged vasculature during tumour growth.

The largest decrease in ΔTRE was observed with LocalNet with an average value of −82.8%, significantly lower than all other methods (figure 6(A), *P*<0.05 for all comparisons). NCC and MI were superior to ICP only in terms of minimising TRE (*P*<0.001) with average ΔTRE of −76.8% and −73.9%, respectively. In terms of shape-based metrics, there was no significant differences between all methods in terms of Dice coefficients (figure 6(B)), although LocalNet provided the highest improvement with 66.5% post-co-registration on average, followed by NCC with 39.1% on average. Similarly, there was no significant differences in average surface distance (figure 6(C)) between all methods, with the greatest average decrease seen with ICP with −9.6%, followed by LocalNet with −7.6%. Only LocalNet provided significantly lower decrease in HD (figure 6(D)) in the longitudinal PDX dataset compared to other methods with −6.7% on average (*P*<0.05 for all comparisons). All other methods resulted in an increase in HD pre vs post-co-registration, meaning that the most distant surface point between paired segmentations got further away post-co-registration.

For intensity-based quality metrics, ΔMI (figure 6(E)) showed no significant difference between methods, with LocalNet providing a marginal 0.77% improvement on average, followed by NCC with 0.58%. ΔSSIM (figure 6(F)) was significantly higher in images co-registered with ICP compared to other methods (*P*<0.05), with an average value of 6.0% followed by LocalNet with 2.8%. Overall, only LocalNet or ICP co-registration was superior (or at least non-inferior) on average to other methods based on evaluated quality metrics in the longitudinal dataset in PDXs. NCC and MI provided improved co-registration by minimising TRE compared to ICP.

## 4. Discussion

*In vivo* mesoscopic PAI has shown great promise in the study of vascular biology in a range of disease settings but image co-registration is challenging due to signal sparsity, the presence of several distinct imaging artefacts, and high sensitivity to experimental conditions. Here, we present an end-to-end testing of curated open-source co-registration methods applied to healthy ear and pathological tumour image pairs at a fixed time-point, and then to tumour image pairs acquired at a series of longitudinal time-points. We compared intensity-, shape- and deep-learning-based methods, demonstrating the first comprehensive testing of co-registration methods for photoacoustic mesoscopy of the vasculature. The ability to overcome technical and biological variability in mesoscopic PAI to align complex vascular networks lays the foundation for monitoring of alterations in the vasculature in preclinical discovery research, for example, in tumour evolution and treatment response assessment *in vivo*, where perturbations can now be assessed and co-localised longitudinally.

Our findings on the single time-point data indicated that tested methods were able to overcome the technical variability of mesoscopic PAI to align 3D vascular networks imaged repeatedly. We found that intensity-based affine co-registration techniques using NCC and MI provide similar image alignment performance. Both outperform shape-based methods using distance-transformed or ICP methods applied on segmented images. Weakly supervised deep learning methods had not been previously reported for 3D vascular imaging co-registration, but showed promise in this study. A weakly supervised U-Net trained to predict deformable registration fields from both intensity and segmentation image pairs, LocalNet, provided accurate co-registration of substructures by optimising subfield deformation throughout the image volume, with similar performance to intensity-based NCC and MI-based methods on the single time-point dataset.

Once the performance of selected algorithms was benchmarked on the fixed time-point dataset, they were further tested on growing PDX tumours where more substantial biological variability is seen as the tumour size and vascularisation increases. Interestingly, on the multi-time-point dataset, the performance of LocalNet was superior compared to other methods. Data acquired on different days showed more substantial changes in imaged vascular structures, which was also impacted by the different positioning of the animals in the imaging system. Since the single time-point dataset was used for training and validation, the longitudinal dataset can be considered as an external unseen testing dataset, hence confirming the performance of LocalNet and demonstrating its generalisability to more complex problems. The overall improvement in quality metrics pre- vs post-co-registration across all selected co-registration methods was lower in the longitudinal dataset compared to the fixed time-point dataset; this finding was expected considering that the visualised anatomy in the latter has not changed between scans, while it has in the former.

Considering that the extent of the vascular volume captured within images acquired on different days was substantively different, conventional iterative one-shot affine image co-registration approaches were not fully able to identify the structures represented in both scans, requiring manual annotations of common structures, whereas learning-based approaches appear be better able to align images. Moreover, the partial overlap of the vascular networks meant that quality metrics assessed on the whole volume of image pairs were confounded by regions not fully represented in both volumes. Hence, though average surface distances were decreased, maximum surface distances (HDs) were increased on average in all methods except LocalNet, in which both metrics improved, in the longitudinal tumour dataset. Therefore, image similarity metrics should be interpreted with care when used for image co-registration.

Prior studies have aligned repeated intensity scans of PAI vasculature acquired at a single time-point and at coarser length-scales than PAI mesoscopy (Yu *et al* 2022, Santi *et al* 2024). Yu *et al* reported that phase correlation, a metric analogous to NCC, combined with MI performed best with a gradient descent-based optimiser framework, akin to the one used in the intensity-based co-registration methods here. Conversely, they were unable to co-register their data successfully using shape-based methods based on image features (Yu *et al* 2022). De Santi *et al* showed that their proposed learning-based co-registration framework adapted from previous work on implicit neural representations (Wolterink *et al* 2022) and integrating multi-scale Frangi vesselness filtering outperformed NCC-based rigid and B-spline deformable co-registration (Santi *et al* 2024). Similarly, we too found that learning-based co-registration can be advantageous, particularly when moving towards analysis of data acquired at different time-points in longitudinal studies.

Segmentation of vascular networks prior to co-registration offers a method to circumvent the impact of confounding factors such as noise and artefacts. Shape-based techniques required segmentation masks while LocalNet additionally required paired intensity images. Segmentation can introduce human bias but here an unsupervised deep learning methodology, VAN-GAN, was used for segmentation (Sweeney *et al* 2024). Leveraging segmentation masks enables more robust alignment, and quality metrics obtained from binary masks appear to better inform on image co-registration quality. In shape-based approaches, ICP gives all the weighting to the surface of the segmented vasculature, where the point cloud and normals are extracted from, which may explain the increased performance from ICP compared to the distance-transformed co-registration approach that emphasises the central line of the vessels for co-registering mesoscopic PAI.

Based on our findings, it would appear that intensity-based affine co-registration techniques are suitable for aligning mesoscopic PAI acquired on a same day or on consecutive days if the anatomy of interest is not changed significantly between scans and if the transducer can be reproducibly positioned to capture the same anatomy. Such methods can be applied in low computational resource settings and require limited programming knowledge, thus showing higher dissemination potential across centres with ranges of expertise. For longitudinal imaging of evolving diseases in which the vasculature is expected to be altered, including for treatment monitoring, we found in this study that manual landmarks identification provide improved initial alignment compared to simple centre-of-mass initialisation. Further, non-rigid learning-based methods, such as LocalNet, provide means to align complex structures altered over time (up to 8-day interval between scans reported in this study). The tested methods can also be extended to other applications such as increasing the imaging field of view in photoacoustic mesoscopy by stitching slightly overlapping sequential scans of a larger skin area of interest, opening new outlooks for imaging larger anatomies. The training of LocalNet required access to computational resources in terms of graphic processing units; even though these have become more widely used by the community, they may not be available across biological laboratories.

Despite our successful implementation of PAI mesoscopic image co-registration, our study has some limitations. First, methods were evaluated on data acquired with a single PAI mesoscopy scanner at a single institution by experienced users. Thus, high data quality might overestimate the performance of the co-registration algorithms when applied to PAI data acquired by different users, disease models, or on different systems, such as clinical scanners (Aguirre *et al* 2017). Although a clear translatability of methods from physiological (murine ears) to pathological (breast cancer xenografts) datasets was observed in our study, it could be expected that in diseases in which endogenous chromophores such as melanin are altered, e.g. in pigmented melanomas (Omar *et al* 2015, He *et al* 2022), the imaged vascular volume will be affected by the absorption in skin layers. Future work should include comparisons on multiple scanners and imaging wavelengths to further assess the impact of data quality on the performance of alignment techniques both preclinically and clinically.

Second, the impact of field of view positioning on the imaged tissue or transducer-to-skin angle were not specifically evaluated. Positioning of the transducer head between scans of the same anatomy is a major source of variability for PAI mesoscopy, considering the top illumination geometry of raster-scanning systems (Hacker *et al* 2023). Moreover, acquisitions can be confounded by cardio-respiratory motion of scanned animals, leading to additional artefacts and discontinuities in the imaged vasculature. In this study, animals were anaesthetised during imaging and the anatomical regions scanned (ear and tumour) enabled repeatable transducer head positioning as both structures protrude from the body of the animal. Limited motion was observed during ear scans, while in tumour-bearing mice, motion was limited by reasonable compression of the tumour with the motorised stage; a motion correction algorithm was applied during image reconstruction to address any residual motion artefact. Using data from regions more greatly confounded by motion, such as those affected by cardio-respiratory motion in the torso, could be investigated in future studies. In clinical PAI mesoscopy studies, scan-to-scan reproducibility for smooth ROI with limited surrounding reference, such as the human skin, might be lower and impact the success of co-registration methods tested in this study.

Third, deep learning-based affine co-registration strategies were not tested. Learning global transformations such as parameterised rigid or affine transformations are sensitive to network initialisation, as for training spatial transformer networks (Jaderberg *et al* 2015), and typically do not converge for one-shot approaches on large 3D datasets such as the ones tested in this study. Learning-based affine transformation might require iterative spatial transformations as described previously (Shen *et al* 2019), but were not tested here. Multiple deep learning affine registration frameworks have been proposed for 3D mono-modal or multi-modal medical imaging alignment, often included as a subnetwork prior to a deformable registration block (Chee and Wu 2018, b, de Vos *et al* 2019, Zhao *et al* 2019, 2020, Mok and Chung 2022). In our study, we favoured centre-of-mass initialisation (fixed time-point dataset) or landmarks initialisation (longitudinal dataset) prior to deformable registration, avoiding the need for a two-step approach and resulting in accurate co-registrations.

Finally, we did not develop specific novel methodologies to tackle the challenge of registering 3D vascular networks in photoacoustic mesoscopy. Future research optimising methods rather than comparing existing ones could provide improved alignment performance on similar datasets.

## 5. Conclusion

We have demonstrated the successful co-registration of vascular networks in photoacoustic mesoscopy in physiological and pathological tissues, acquired at a single time-point and at multiple time-points. Overall, a weakly supervised learning-based method, LocalNet, provided similar co-registration performance across single time-point datasets compared to other non-deep learning intensity-based methods. LocalNet was superior based on TRE measures in the longitudinal dataset, where the imaged vasculature was altered by the disease process between imaging time-points. The compared methods overcame the technical scan-to-scan variability, suggesting that quantifiable residual differences post-co-registration could be attributed to disease mechanisms for biological discovery. In fact, these findings indicate the feasibility of co-locating vascular changes over time through mesoscopic PAI co-registration for the longitudinal monitoring of disease processes and potentially of therapeutic response. Users should assess the extent of expected changes of the imaged vasculature in their biological problem of interest to select which type of 3D alignment method to employ for co-localising vascular changes over time with PAI. Thus, by demonstrating the feasibility of co-registering photoacoustic mesoscopy images, we have taken a step towards enabling longitudinal characterisation of complex vascular biology in evolving pathologies, such as cycling hypoxia in cancer, response to therapy including chemo- and radiotherapy, and for the monitoring of diseases beyond cancer, including skin conditions such as psoriasis.

## Data Availability

The data that support the findings of this study are openly available under the CC-BY 4.0 license at the following DOI: https://doi.org/10.17863/CAM.111991. The code developed and used in this study is available under the MIT license at the following URL: https://github.com/BohndiekLab/coregistration-longitudinal-pai-mesoscopy.
